# Roads to Veterinary Education

**Published:** 1894-12

**Authors:** 


### ROADS TO VETERINARY EDUCATION.

We have come to the cross-roads in veterinary college work in America, and night falls upon the longer existence of short-term schools, and the setting sun has closed over the forced plants which ripened as graduates from many of our veterinary colleges. Which road will you travel? is now the serious question of the hour. The signs of the times are clear as a noonday sun; the lesson learned in the present hour is painful to observe. Of all the schools, few have so well prepared their graduates as to be able to pass the necessary civil service examination as Meat Inspectors under the National Government. The country from Maine to California is flooded with members of the profession, clothed with sheepskins of varied worth, and hundreds of whom are to-day not making a decent living; hosts of others are turning their attention to other professions and occupations; while others are yielding to unscrupulous methods of earning a living, and bringing disgrace upon themselves and just criticism of the profession. What a responsibility to undertake, for any body of men to establish a college whose work is to live after them for many years to reflect their errors, imperfect work and lack of appreciation of the great trust they thoughtlessly accepted. Not a single two years' school to-day but must face a serious future from this time forth, and failure to recognize the gravity of the situation will determine the closing of other doors. There must be no waiting longer for what some other school is going to do, for only those who quickly meet the present issue will survive the present forcible reminder of the character, quality and scope of work they have been doing.

With perhaps the one notable exception, now filled by the establishment of the California Veterinary College as a department of the State University, there is no need of a single new school in our land, and no longer a place for some of those which now exist. Without the need of special capital; without the requirements of a large plant; without the accessories of even many of the trades to-day, why is it that so many of our calling are without support and sustenance such as a profession of so much importance should insure? The answer quickly follows, because so few of our number are properly equipped for the work that is theirs to do. Without preparatory training, without laboratory work, lack of fitting surgical instruction, filled to overflowing with undigested book knowledge, and long-drawn lectures of glittering generalities forced upon students in the short-term courses, has now solved its own fate, and which road will you travel? is now pressing for an answer.

